# Autoimmune Neurological Disorders with IgG4 Antibodies: a Distinct Disease Spectrum with Unique IgG4 Functions Responding to Anti-B Cell Therapies

**DOI:** 10.1007/s13311-022-01210-1

**Published:** 2022-03-15

**Authors:** Marinos C. Dalakas

**Affiliations:** 1 Department of Neurology, Thomas Jefferson University, Philadelphia, PA USA; 2 Neuroimmunology Unit National and Kapodistrian University of Athens Medical School, Athens, Greece

**Keywords:** IgG4-autoimmune neurological diseases, IgG4 antibodies to nerve antigens, complement activation, FcγRIIb receptors, anti-B cell therapies, IVIg

## Abstract

The main IgG4 antibody–mediated neurological disorders (IgG4-ND) include MuSK myasthenia; CIDP with nodal/paranodal antibodies to Neurofascin-155, contactin-1/caspr-1, or pan-neurofascins; anti-LGI1 and CASPR2-associated limbic encephalitis, Morvan syndrome, or neuromyotonia; and several cases of the anti-IgLON5 and anti-DPPX-spectrum CNS diseases. The paper is centered on the clinical spectrum of IgG4-ND and their immunopathogenesis highlighting the unique functional effects of the IgG4 subclass compared to IgG1-3 antibody subclasses. The IgG4 antibodies exert pathogenic effects on their targeted antigens by blocking enzymatic activity or disrupting protein–protein interactions affecting signal transduction pathways, but not by activating complement, binding to inhibitory FcγRIIb receptor or engaging in cross-linking of the targeted antigen with immune complex formation as the IgG1-IgG3 antibody subclasses do. IgG4 can even inhibit the classical complement pathway by affecting the affinity of IgG1-2 subclasses to C1q binding. Because the IgG4 antibodies do not trigger inflammatory processes or complement-mediated immune responses, the conventional anti-inflammatory therapies, especially with IVIg, immunosuppressants, and plasmapheresis, are ineffective or not sufficiently effective in inducing long-term remissions. In contrast, aiming at the activated plasmablasts connected with IgG4 antibody production is a meaningful therapeutic target in IgG4-ND. Indeed, data from large series of patients with MuSK myasthenia, CIDP with nodal/paranodal antibodies, and anti-LGI1 and CASPR2-associated syndromes indicate that B cell depletion therapy with rituximab exerts long-lasting clinical remissions by targeting memory B cells and IgG4-producing CD20-positive short-lived plasma cells. Because IgG4 antibody titers seem reduced in remissions and increased in exacerbation, they may serve as potential biomarkers of treatment response supporting further the pathogenic role of self-reacting B cells. Controlled trials are needed in IgG4-ND not only with rituximab but also with the other anti-B cell agents that target CD19/20, especially those like *obexelimab* and *obinutuzumab*, that concurrently activate the inhibitory FcγRIIb receptors which have low binding affinity to IgG4, exerting a more prolonged anti-B cell action affecting also antigen presentation and cytotoxic T cells. Antibody therapies targeting FcRn, testing those anti-FcRn inhibitors that effectively catabolize the IgG4 antibody subclass, may be especially promising.

**Supplementary Information:**

The online version contains supplementary material available at 10.1007/s13311-022-01210-1.

## Introduction

The IgG4 subclass of autoantibodies has been associated with a broad spectrum of more than 12, multisystemic or fibroinflammatory autoimmune disorders, referred to as IgG4-related diseases (IgG4-RD). These disorders, insidiously and sometimes subclinically, affect lacrimal and salivary glands, thyroid, lungs, bile ducts, kidneys, pancreas, aorta, retroperitoneum, and orbits in a form of orbital myositis [1–5]. They are generally poorly understood because only a few of them, like pemphigus vulgaris, membranous nephropathy, and thrombotic thrombocytopenic purpura, are characterized by disease and tissue-specific autoantibodies [1, 5]. In contrast, the IgG4 neurological disorders (IgG4-ND) are now becoming an immunopathologically distinct disease spectrum, as recently pointed out [1], because of their association with pathogenic IgG4 antibodies targeting neural-specific antigens. The IgG4-ND include the following: (a) MuSK myasthenia; (b) CIDP with paranodal antibodies to Neurofascin-155, contactin-1, CASPR1, and nodal/paranodal pan-neurofascins (NF140/NF186/NF155); (c) LGI1 or CASPR2-associated autoimmune CNS disorders and peripheral nerve pain syndromes presented as encephalitis, autoimmune epilepsy, Morvan syndrome, neuromyotonia, or autoimmune pain; (d) the anti-IgLON5 disorder, a rare CNS disease spectrum with multiple manifestations; and (e) several cases of anti-DPPX encephalitis, characterized by gastrointestinal symptoms, cognitive dysfunction, and neuronal excitability, as discussed later [1, 5–10].

In contrast to their IgG1-3-associated counterparts, the IgG4-ND exhibit most of the times poor long-term response to IVIg and inadequate long-term response to steroids or plasmapheresis, but excellent response to anti-B cell therapies, like rituximab [1, 4]. It has now become apparent that many of these patients clinically present similarly to their IgG1-3-associated identical syndromes and they are almost always treated with conventional immunotherapies of steroids, IVIG, plasmapheresis, and oral immunosuppressants until recognized in retrospect that they do not adequately respond, questioning not only the diagnosis but also the associated autoimmunity [1]. The need to appreciate why these patients respond predominantly to anti-B cell therapies is important for the clinical neurologists to initiate the proper immunotherapy early in the disease course to avoid therapeutic delays [1]. In addition to IgG4-ND, the IgG4-associated systemic diseases are also of interest to neurologists because they not only do cause various multiorgan, fibroinflammatory, or lymphoproliferative conditions, but also can exhibit neurological symptomatologies highlighted by hypertrophic pachymeningitis, hypophysitis, and orbital myositis due to chronic meningeal and orbital muscle involvement [2, 3].

The paper is a detailed extension of the recently published review on the same topic which was focused on why the patients with IgG4 subclass of antibodies do not respond to IVIg [1]. The present review is centered on the clinical spectrum, immunopathogenesis, and therapies of IgG4-ND, especially focused on the uniqueness of the IgG4 subclass and the mechanisms by which the IgG4 antibodies disrupt their targeted antigens in all IgG4-ND compared to IgG1-3 subclasses; highlights the activation of plasmablasts in IgG4 production supporting the role of rituximab in early therapy initiation; addresses the potential role of IgG4 antibodies as biomarkers for disease remissions or the need for repeated therapies; explains briefly the ineffectiveness of IVIg and conventional immunotherapies in inducing long-term remissions; and elaborates on the rationale of new antibody therapies more tailored to targeting IgG4 as the most suitable options in treating these disorders.

## IgG4 Neurological Autoimmune Diseases and Effects of IgG4 on Neural Antigens

The IgG4 antibodies arise after chronic antigenic exposure late in the immune response and, after undergoing several rounds of affinity maturation and somatic hypermutation, they exhibit very high affinity for their target antigen [5, 11]. In contrast to IgG4-RD however which have a broad and heterogeneous multi-organ clinicopathology with tumefactive lesions and IgG4 + plasma cell infiltrates in multiple tissues [2–4], the IgG4-ND exhibit distinct signs of antigen-specific CNS or PNS neuro-autoimmunities characterized by neural cell–specific antibodies exerting pathogenic effects on their targeted antigens. Most importantly, in IgG4-ND, the antibodies do not cause an inflammatory-mediated tissue destruction as the IgG1-3 subclass of antibodies do, but inhibit cellular adhesion, block enzymatic activity, or disrupt protein–protein interactions affecting signal transduction pathways [1, 5, 11]. This is also in contrast to the IgG4-RD where only a few of the IgG4 antibodies are potentially pathogenic, mostly the anti-M-type phospholipase A2 receptor 1 and thrombospondin type-1 in membranous nephropathy, the anti-desmoglein in pemphigus vulgaris, and the anti-metalloprotease ADAMTS13 in thrombotic thrombocytopenic purpura [1–5, 11]. The main IgG4-ND include [1] :

1. Anti-MuSK-MG

About 7% of all AChR-negative MG patients have IgG4 antibodies against muscle-specific kinase (MuSK), a post-synaptic transmembrane polypeptide expressed at the neuromuscular junction. MuSK, via interactions with agrin and rapsyn, plays a fundamental role in AChR clustering which is essential for efficient neuromuscular transmission [5, 6, 12]. When agrin is released from the presynaptic nerve terminal, it binds to low-density lipoprotein receptor 4 (Lrp4), which then binds to MuSK leading to MuSK activation and clustering of AChR on top of post-synaptic folds [12]. MuSK antibodies are of the IgG4 subclass and they are pathogenic causing dysfunction of the neuromuscular junction by interfering with AChR clustering through inhibition of Lrp4/MuSK signaling, but not by antigen cross-linking, internalization, and end-plate destruction as the common IgG1-AChR antibodies do [5, 6, 12]. MuSK IgG4 can passively transfer disease and the antibody titers correlate with disease severity being reduced when patients are in remission [1, 12–14], as discussed later.

Although patients with MuSK-MG may have a phenotype similar to AChR-MG, they most often have unique presentation with selective weakness and atrophy of the neck, tongue, shoulder, and bulbar muscles [12–14]. In MuSK-MG, the thymus lacks the histological alterations seen in AChR-MG and thymectomy is not needed; furthermore, anticholinesterases are ineffective or may even worsen the disease [13].

2. CIDP with paranodal antibodies

A breakthrough in CIDP antibody autoimmunity has been the remarkable observation that a subset of patients who do not respond to IVIg or plasmapheresis have IgG4 antibodies to nodal/paranodal antigens directed against neurofascin-155 (Nfasc155), neurofascin-140/186 (Nfasc140/186), contactin-1 (CNTN1), and contactin-associated protein 1 (Caspr1) [1, 7, 15–19]. These IgG4 antibodies form a clinicopathologically distinct CIDP subset, comprising 10% of all CIDP patients, often referred to as autoimmune nodopathies [20]. Most commonly, CIDP nodopathies are associated with anti-Nfasc155, followed by Caspr1 and CNTN1, and rarely against both Nfasc140/186 and Nfasc155 [1, 7, 15–22]. In contrast to common CIDP characterized by macrophage-mediated demyelination, complement activation and myelin destruction, in patients with CIDP nodopathy, except of rare exceptions, the IgG4 nodal/paranodal antibodies do not cause macrophage activation and demyelination, do not fix complement to internalize specific nodal/paranodal antigens, and do not elicit an inflammatory response; instead, they affect protein-protein interaction exerting a functional blockade by disrupting the paranodal axoglial contact leading to conduction failure [1, 7, 18–21].

NF155 is a Schwann cell adhesion protein that, at the paranodal terminal myelin loops, interacts with CNTN1/Caspr1 complex anchoring myelin to axon, forming a protein complex critical for maintaining the integrity of the nodal structures ensuring rapid impulse propagation [18–21]. The antibodies against these proteins are pathogenic by binding distinct epitopes associated with cell adhesion, disrupting the NF155/CNTN1 components by affecting glycosylation and sodium currents or dissecting the axoglial junction resulting in disturbed conduction [7, 15–22]. Based on passive transfer experiments, anti-Nfasc155 IgG4 and anti-CNTN1 IgG4 cause paranodal disorganization but act differently. In contrast to anti-CNTN1 that cause functional blockade by inhibiting the interaction of Nfasc155 with the CNTN1/CASPR1 complex dismantling the paranodal axoglial contact, the anti-Nfasc155 IgG4 seems to bind to the Schwann cell surface causing Nfasc155 aggregation and depletion [15–22]. Furthermore, anti-CNTN1 autoantibodies can cause reduction in contactin-1 surface expression on dorsal root ganglionic and cerebellar neurons and decrease the sodium currents in the dorsal root ganglionic cells without affecting the sodium channel density, providing a pathophysiologic correlate of sensory ataxia often seen in these patients [21]; the antibody subclass in this study was not however specified.

The autoimmune nodopathy patients may have the typical clinical phenotype of CIDP but most often have distinct characteristics highlighted by subacute onset of severe neuropathy, tremor, and sensory ataxia. Most importantly, and in contrast to their IgG1-3 counterparts, they respond poorly to IVIg and plasmapheresis but excellently to rituximab that induces long-lasting remissions, necessitating increased awareness to identify them from the outset [1, 7, 15–21]. Because subclass switch from IgG1-3 to IgG4 can also occur, as discussed later, an IgG4 nodopathy/paranodopathy should be considered when a CIDP patient previously responding to IVIg becomes IVIg unresponsive [1].

3. LGI-1 and CASPR2 autoimmune syndromes

Leucine-rich glioma-inactivated-1 (LGI1) and contactin-associated protein 2 (CASPR2) function to stabilize the voltage-gated potassium channel complex into the membrane [8, 9]; LGI1, in particular, plays a key role in bridging the pre-synaptic voltage-gated potassium channel protein Kv1.1 with the post-synaptic AMPA receptor through interaction with synaptic anchor molecules ADAM22/23. The anti-LGI1 IgG4 antibodies alter the binding of LGI1 with ADAM22 and decrease the post-synaptic levels of AMPA receptors [8]. Since LGI1 is also involved in cell adhesion probably through an interaction with Nogo receptor-1 [5], these antibodies may cause disadhesion of the targeted antigen similar to what was described above in CIDP nodopathies [17, 19]. The LGI1 antibodies are mostly of the IgG4 subclass as demonstrated by staining on transiently transfected cells [5], but some of them may be also of the IgG1 and IgG2 subclass. Of interest, and adding some complexity, an IgG4 anti-LGI1 antibody targeting a specific LGI1 epitope, was experimentally shown to cause internalization of the LGI1/ADAM complexes,suggesting a potentially monovalent mechanism which was surprising because IgG4 molecules, as discuss later, Fab-arm exchange lacking bivalency [23]. Although such a rare possibility remains to be investigated, antibody-antigen internalization and antigen cross-linking are mediated by the bivalent IgG1-3 antibody subclasses and not by the monovalent IgG4 subclass. The Caspr2 autoantibodies, also mostly of IgG4 subclass, do not affect the surface expression of Caspr2 and do not cause internalization of Caspr2 but inhibit its interaction with its binding partner contactin-2 resulting in functional blocking of cell adhesion molecular interactions [24].

LGI1 and CASPR2 are expressed in both the CNS and PNS, including peripheral nerves and dorsal root ganglia. Sera from patients with CASPR2—but not LGI1—antibodies bind in vitro to unmyelinated human sensory neurons and rodent dorsal root ganglia [25], suggesting peripheral pathophysiological differences among the two that impact on their clinical symptomatology especially the association with neuropathic pain, as recently highlighted [25]. Increased titers of LGI1 antibodies are also present in the serum and CSF.

Although patients with anti-LGI-1 and CASPR2 antibodies have clinical heterogeneity, they also exhibit significant overlapping clinical symptomatology; the antibodies to LGI1 are most commonly associated with limbic encephalitis and epilepsy while the antibodies to CASPR2 with Morvan syndrome, neuromyotonia, and neuropathic pain [1, 5, 8, 9, 25]. Refractory epilepsy, and mental and behavioral abnormalities are variably present. Pain, as seen in small fiber sensory neuropathy with reduced intraepidermal nerve fiber densities and autonomic nervous system features like seen in POTS, is now increasingly recognized in CASPR2-positive patients forming a well-defined subset of autoimmune pain syndromes responding to immunotherapies [23]. Seizures involving ipsilateral face and upper limb dystonia (faciobrachial dystonic seizures, FBDS) can be distinct manifestations in LGI1-associated encephalitis.

4. Anti-IgLON5 disorder

IgLON5 is a neuronal cell adhesion molecule attached to the cell membrane by a glycosyl-phosphatidylinositol anchor protein, known to play crucial roles in cell adhesion and signaling by interacting with other cytoskeletal proteins [10]. Anti-IgLON5 antibodies cause disorganization of the cytoskeleton in cultures of rat hippocampal neurons producing dystrophic neurites, axonal swellings and decrease of IgLON5 clusters on neuronal surface, all abnormalities similar to those seen in hypoxic conditions or in early stages of neurodegeneration. It has been postulated that IgLON5 antibodies disrupt the crosstalk between cell surface and cytoskeleton leading to abnormal accumulation of neurofilaments, providing a link between antibody-mediated autoimmunity and neurodegeneration [26]. The antibodies against IgLON5 are most of the times of the IgG4 subclass, as confirmed by immunostaining of rat hippocampus [26].

Anti-IgLON5 antibodies define a complex syndrome of chronic progressive brainstem symptomatology, gait instability, distinct non-rapid eye movement (REM) and REM parasomnias, obstructive sleep apnea, sleep-disordered breathing, cognitive decline, and movement disorders as recently identified, most commonly craniofacial dyskinesias, dystonia, chorea, and abnormal eye movements [27, 28]. Multiple movement disorder in a setting of sleep alterations, bulbar dysfunction, and cognitive impairment should raise suspicion of this still evolving disease spectrum [28].

5. Anti-DPPX disorder

These patients have antibodies to dipeptidyl peptidase**–**like protein (DPPX), a regulatory subunit of neuronal Kv4.2, rapidly inactivating potassium channel complex, responsible for transient inhibitory currents that regulate repetitive firing rates into neuronal dendrites. The DPPX antibodies are both of the IgG4 and IgG1 subclass and reduce cell membrane protein levels of DPPX and Kv4.2 potassium channels in cultured neurons increasing neuronal excitability in myenteric neurons [29]. Because of the widespread distribution of Kv4.2 complexes, these patients present with a multifocal neurologic phenotype with severe prodromal weight loss or diarrhea, followed by cognitive dysfunction, memory deficits, CNS hyperexcitability, hyperekplexia, myoclonus, tremor, seizures, dysautonomic manifestations, and brainstem or cerebellar dysfunction, peaking at 8 months from onset [29–31]. The triad of weight loss due to gastrointestinal symptoms, cognitive-mental dysfunction, and CNS hyperexcitability is highly suspicious for DPPX autoimmunity. Patients respond to immunotherapy with reversibility of the effects exerted by these antibodies in cultured neurons. The DPPX-associated neurologic syndrome resemble that of PERM [31] and, although the antibody effects are probably due to a combined effect of IgG1 and IgG4 immunoglobulins, it responds better to second-line immunotherapy most often with rituximab [29, 30]; interestingly, relapses are more frequent when this therapy is discontinued pointing more to an IgG4 rather than IgG1-connected immunopathogenesis.

## The Distinct Anti-Inflammatory Functions of IgG4 and the Factors Contributing to IgG Subclass Switch

In healthy adults, the IgG4 is the least common IgG subclass, comprising less than 5% of the total IgG with concentration ranging from 0.08 to 1.4 g/l [2–4]. The IgG4 antibodies have evolved as an anti-inflammatory response to alleviate IgE-mediated allergic inflammation [2] and have unique structure compared to the other subclasses.

**Fig. 1:**
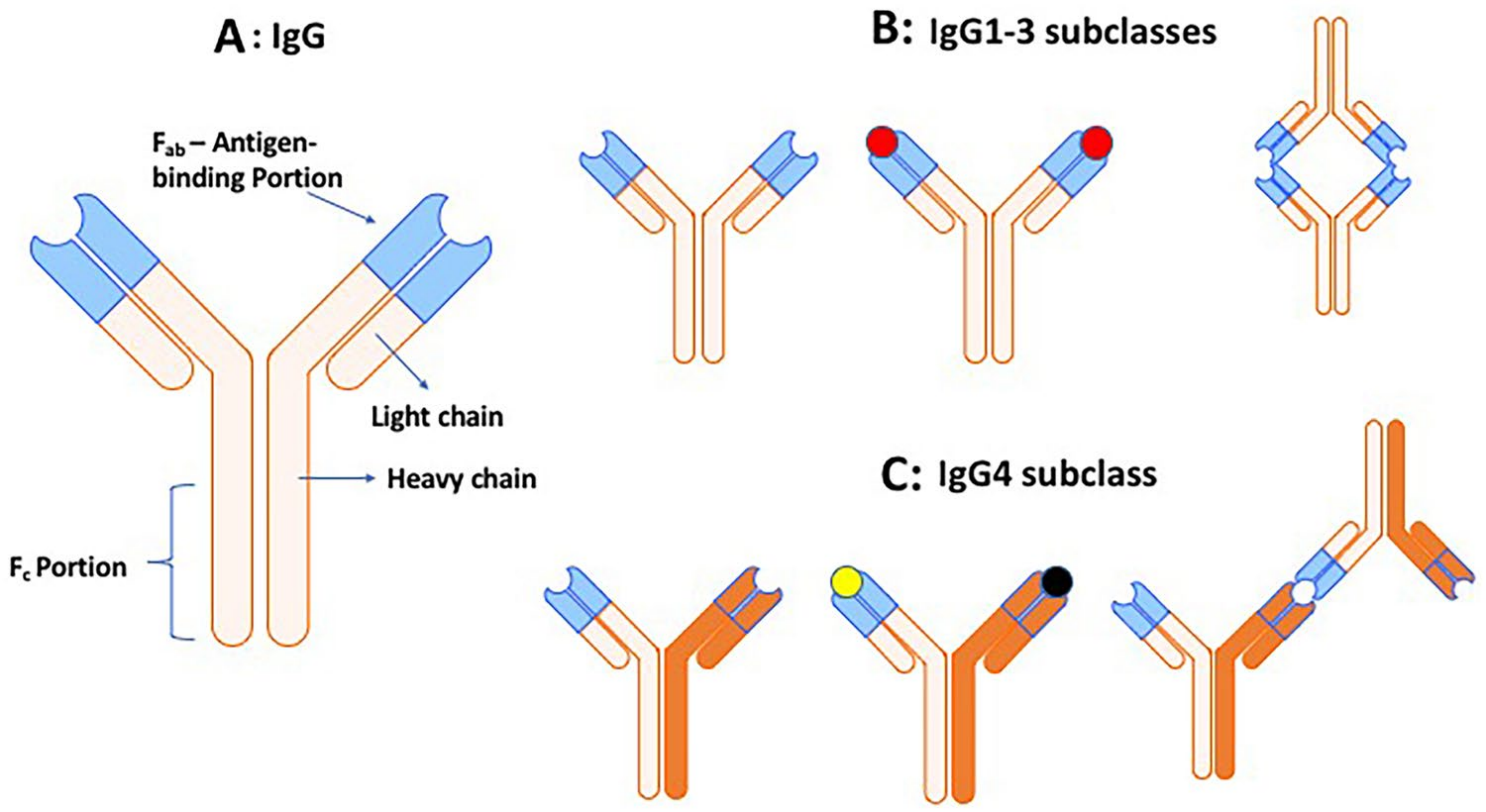
**A** The IgG has two heavy and two light chains, both with a constant fragment (Fc), identical for all antibodies of the same isotype which binds to the cell surface allowing phagocytosis. IgG has two antigen-binding fragments (Fab) that bind to a specific antigen. **B** The IgG1-3 antibody subclasses have two identical antigen-binding sites (in blue) binding to the same antigen (depicted both in red) with both arms; they are therefore *bivalent* and *monospecific*. **C** The IgG4s have unique structure in the hinge region, and their two heavy and light chains are joined by non-covalent bonds; as a result, they cannot cross-link identical antigens as the IgG1-3 do (depicted one in black and the other in yellow). Instead, the IgG4s continuously undergo half-antibody “Fab-arm exchange” recognizing the same antigen only with one arm; they are therefore functionally *monovalent* and *bispecific*

The IgG has two heavy and two light chains, both with a constant fragment (Fc) identical for all antibodies of the same subclass by which bind to cell surface allowing phagocytosis, and two antigen-binding fragments (Fab) that bind to a specific antigen (A, in Fig. 1). The IgG1-3 antibodies have two identical antigen-binding sites binding to the same antigen, being *monospecific* and *bivalent* (B in Fig. 1). In contrast, the IgG4s are derived from several residues in the CH2 and hinge region and have their two heavy and light chains joined by non-covalent bonds (C in Fig. 1); because of such a unique structure, the IgG4s cannot cross-link identical antigens, but they continuously undergo half-antibody “Fab-arm exchange” recognizing the antigen only with one arm, being *functionally monovalent* and *bispecific *(Fig. 1). By recognizing the antigen only with one Fab-arm, the IgG4 cannot result in high concentration of antigen-bound molecules at their targets [32]. The consequential effects of these properties are highlighted by two unique binding peculiarities of IgG4 compared to the IgG1 subclasses; first, they cannot bind to C1q complement component to activate the complement cascade and, second, bind deferentially to Fc receptors having reduced capacity to bind to inhibitory Fcγ receptor (FcγRIIB) but enhanced capacity to bind to activating FcγRIA/FcγRIIA receptors [1–5]. As a result, the IgG4 antibodies have non-inflammatory properties being unable to form cross-linked immune complexes to internalize and degrade their targeted antigen and inadequate to activate cellular or complement-mediated immune responses [1–5, 32]. In contrast to the other IgG subclasses, they exert pathogenicity by mechanical blocking of protein–protein interactions interfering directly with the function of the antigen or by affecting signal transduction pathways [1, 2, 5].

Regarding the effect of all IgG subclasses on complement, C1q binds more efficiently and strongly to IgG3 followed by the IgG1 subclass while barely interacts with IgG2 and has no affinity to IgG4. Importantly, IgG4 not only is unable to bind C1q but there is evidence that can also inhibit the first steps of the complement classical pathway by affecting the affinity of IgG1 and IgG2 subclasses to C1q binding, but without exerting any inhibitory impact on the affinity of IgG3 and IgM to CIq binding [33]. Although there is no evidence that IgG4 has the potential to activate complement, recent in vitro data suggest that if IgG4 if aberrantly glycosylated, it can activate complement via the lectin pathway based on an experimental model of membranous nephropathy [34]. Whether aberrant glycosylation of IgG4 can happen in vivo and be substantial enough to trigger complement activation of clinical significance in IgG4-ND remains an unproven possibility.

Based mostly from IgG4-RD observations, the production of IgG4 is facilitated by IL-10 which plays a key role in switching the B cell’s isotype to IgG4 subclass [2–4]. Follicular helper T (Tfh) cells that produce IL-4, IL-10, and IL-21 along with the IL-10-expressing T-follicular regulatory cells also participate in class switch [1–4, 32, 35]. The IgG4 antibody production is additionally facilitated by the B cells as their role of serving as antigen-presenting cells that activate CD4 + cells, undergoing serial rounds of proliferation [3]; this notion is supported by the decline of CD4 + cytotoxic T cells after anti-B cell-therapy and their increase during disease activity [2–4]. Circulating memory B cells and plasmablasts are also increased in patients with IgG4-RD with prominent IgG4-producing CD19flCD20** + **CD27hiCD38hi plasmablasts during relapses [2–4].

## The Need to Identify an Insidious IgG4 Subclass Switch

Although immunoglobulin subclass switch can often occur late in the immune response due to the dynamic process of maturation and hypermutation, in IgG4-ND, an insidious subclass switch from IgG1-3 to IgG4 may have clinical consequences regarding response to therapies especially to IVIg [1] because a patient previously responding to IVIg may become IVIg-unresponsive if is sub-classed to IgG4. This has been already noted in a nodal CIDP patient, who was switched from IgG3 against CNTN1/CASPR1 to IgG4 against CASPR1 and stopped responding to IVIg [36]; the reverse has occurred in a MuSK-MG patient where a switch from IgG4 to IgG1 anti-MuSK antibodies was associated with stable remission [37]. Vigilance is therefore needed when a previous IVIg-responsive patient does not anymore respond [1]. The IgG4 subclass may be also considered when enrolling IVIg-responding or non-responding patients with CIDP or MG in future randomized trials. Subclass switch may be especially relevant in LGI-1 and CASPR2 as well as in DPPX autoimmunities because some of these patients have from the outset antibodies either of the IgG1-3 or of the IgG4 subclass and a potential subclass switch may be more likely to occur due to chronic antigenic stimulation.

## Understanding Treatment Efficacies and Therapeutic Strategies in IgG4-ND

### The Ineffectiveness of IVIg in IgG4-ND

A series of observations, especially in CIDP patients with paranodal antibodies, have consistently shown that these patients did not respond from the outset to conventional therapies with IVIG, which is the treatment of choice for CIDP based on controlled studies, generating dilemmas even about the diagnosis [1, 7, 20, 22]. Similar has been the experience with MuSK-MG which, in contrast to AChR-MG, also responds inadequately to IVIg but robustly to rituximab. Based on the mechanistic effects of the IgG4 and the modes of action of IVIg, it is now apparent not only why IVIg is ineffective but also why the conventional therapies with plasmapheresis and immunosuppressants, but even many times with steroids, offer suboptimal efficacy for long-term remission, in spite of early benefits in some of them. As mentioned earlier and recently highlighted [1], in IgG4-ND, there is no antigen cross-linking or immune complex formation, no recruitment of immune cells via Fc receptors, and no antigenic destruction via phagocytosis or antibody-dependent cellular cytotoxicity; because it is these very specific inflammatory processes which are inhibited by IVIg and characterize its mode of action and its remarkable efficacy in IgG1-ND [38, 39], the fundamental beneficial effects of IVIg are irrelevant to IgG4 pathogenicities [1]. The main immunoregulatory and anti-inflammatory effects of IVIg that define its effectiveness in IgG1-3 antibody subclass, but contrast with their irrelevance and ineffectiveness in IgG4-ND, as recently elaborated [1], are summarized below:

i) *Neutralization of pathogenic autoantibodies by idiotypic antibodies of the IgG1subclass*. The IVIg preparations contain minimal IgG4 (from 0.7 to 2.6%) because more than 95% of IgG is of the IgG1 subclass. IVIg, derived from thousand donors, contains anti-idiotypic IgG1 antibodies forming dimers connected between their F(αβ^′^)_2_ domains (see B in Fig. 1) that bind and neutralize the patients’ pathogenic autoantibodies [38–42]. This effect has been shown in demyelinating neuropathies with anti-GM1 or other anti-glycolipid antibodies [43, 44] where the IgG1 idiotypes within the IVIg inhibit or neutralize the blocking impulse propagation signals exerted by these patients’ sera, resulting in quick clinical benefits [1, 43–45]. Since IVIg does not contain idiotypes of the IgG4 subclass, it cannot exert similar neutralizing effects on IgG4 antibodies in IgG4-ND [1].
ii) *Inhibition of complement binding, preventing the formation of membranolytic attack complex (MAC)*. IVIg inhibits complement uptake by forming covalent or non-covalent bonds with C3b, intercepting the formation and deposition of MAC by the complement-fixing IgG1 antibodies on the targeted tissues preventing antibody-mediated cytotoxicity [38–40, 46, 47]. This key anti-complement effect is not applicable to IgG4 because these antibodies do not fix complement.
iii) *Upregulation of the inhibitory FcγRIIB receptors*. IgG molecules bind through their Fc region to various FcγR on monocytes, macrophages, dendritic cells, and B cells and exert either activating signaling via the FcγRIA, FcγRIIA, and FcγRIIIA or inhibitory signals via the FcγRIIB, mediating inflammatory and immune effector functions including cellular activation and cytokine production [38–40, 48–51]. IVIg selectively upregulates the inhibitory FcγRIIB and inhibits phagocytosis and cytokine production intercepting antibody-dependent cell-mediated cytotoxicity [38–42]. CIDP patients have lower than normal FcγRIIB on B cells and monocytes but after IVIG, there is FcγRIIB upregulation coinciding with clinical improvement even predicting response to IVIG [38–40, 48–51]. This important effect is not applicable to IgG4-ND because IgG4 antibodies cannot bind to inhibitory FcγRIIB receptor [1–3].
iv) S*uppression of pathogenic cytokines and immunoinflammatory molecules*. IVIg effectively suppresses pro-inflammatory cytokines [38–40] controlling immune activation. These effects are irrelevant to IgG4-ND because IgG4, being anti-inflammatory, does not recruit immune cells via Fc receptors or induces phagocytosis not triggering tissue inflammation [1–3].
v) *Saturation of the FcRn receptors, enhancing catabolism of IgG1-3 antibody subclass*. IgG antibodies bind to FcRn to return intact back into the circulation, being protected from degradation by the lysosomes [52]. The supraphysiological levels of IgG derived from IVIg infusions saturate the FcRn so a portion of the endogenously produced pathogenic IgG antibodies are not recycled back to the circulation but degraded [52, 53], reducing the circulating pathogenic autoantibodies by up to 40% [54]. IVIg is likely not sufficiently effective in IgG4 recycling, as is for IgG1-3 subclasses, although this has not been explored.

### The Rationale of Rituximab as Targeted Therapy: Experience from IgG4-RD

Because in IgG4-RD, the B cells are activated and expanded and the IgG4 antibodies correlate with disease activity, B cell depletion therapy with rituximab is a reasonable therapeutic target [1–4]. This also applies to IgG4-ND because, although different from IgG4-RD, the underlying mechanism of IgG4-induced autoimmunity is similar and the IgG4-producing subpopulation of B cells is a common target. Rituximab, is a chimeric monoclonal antibody that targets CD20, present on all B cells, except stem cells, pro-B cells, and plasma cells, and depletes circulating B cells but not the bone marrow and lymph node B cells [55, 56]. Although immunoglobulins are produced by long-lived plasma cells, the IgG4 antibodies are likely produced by CD20-positive short-lived plasma cells stimulated by IL-4 and IL-21 [2–4, 57, 58]. Because rituximab eliminates B cells before they differentiate into plasma cells, it can effectively target the IgG4-producing CD20-positive short-lived plasma cells and their related CD20 + precursors, reducing both the number of plasmablasts and the serum IgG4 levels compared to IgG1 [2–4, 57, 58].

A number of non-randomized trials, summarized from 105 articles, have shown that rituximab is effective in patients with IgG4-RD who do not respond or have become refractory to corticosteroids [3, 4]. In those IgG4-RD patients with high disease activity or relapses, the circulating IgG4-producing memory B cells and the CD19**fl**CD20**_**CD27hiCD38hi plasmablasts are increased and oligoclonally proliferate [3, 4, 58] but after induction therapy with rituximab, they are reduced, coinciding with clinical improvement [4]. Most IgG4-RD patients were able after rituximab to discontinue glucocorticoids and other immunosuppressants but required maintenance therapy to lower the risk of relapses [4]. The rituximab biosimilar CT-P10 (TruximaTM) was shown to be equally effective [4].

### Experience with Rituximab in IgG-4-ND

The effect of anti-B cell therapies in IgG4-ND is very similar to IgG4-RD but even more compelling based on larger but also uncontrolled series, as summarized below for each disorder:

- *Musk-MG*. In a multicenter, blinded, prospective review, 14/24 (58%) of patients treated with rituximab reached the primary outcome after a median follow-up of 3.5 years, compared to 5/31 (16%) of controls (*p* = 0.002) [59]. In addition, 29% of rituximab-treated patients required a mean prednisone dose of 4.5 mg/day compared to 13 mg/day required by 74% of controls (*p* = 0.005) [58]. In another study, the IgG4-MuSK antibodies were markedly reduced 2–7 months after rituximab being even undetectable within 2 years, coinciding with several years of clinical remission and sustained improvement [60]; in one patient in this series who did not respond, the MuSK-IgG4 antibodies remained unchanged, supporting the view that short-lived antibody-secreting CD20 + cells are the main producers of MuSK antibodies [60, 61]. Similar has been our experience. We have been personally following 3 anti-MuSK-MG patients for 10–25 years, all requiring plasmapheresis, IVIg, CellCept, and high-dose prednisone and one of them frequent hospitalizations; after one only rituximab infusion, all three have become neurologically normal maintained on low, 12–15 mg of prednisone every other day. Although this is arguably an anecdotal tiny series, having experienced the difficulties these patients had for many years with severe relapses and emergency hospitalizations, it is difficult not to be impressed with such an outcome. It is therefore compelling to conduct controlled trials in large series.
- *Neurofascin-155 and CASPR1/CNTN1 CIDP*. The evidence that these patients are unresponsive to IVIg has been overwhelming and almost a hallmark of autoimmune nodopathies, as more than 90% of these patients have been originally diagnosed as CIDP-unresponsive to IVIG based on many reports [7, 15–20]. A recent large international series re-confirmed that more than 80% of these patients respond to rituximab [20]. Today, rituximab should be the treatment of choice from the outset in autoimmune nodopathy patients to prevent irreversible deficits, although controlled studies are clearly needed. There is however also evidence based on uncontrolled series that some CIDP patients with IgG1-3 antibodies also respond to rituximab. The ineffectiveness to IVIG was also confirmed in a recent retrospective series of 214 CIDP patients, 6.5% of which were NF155-IgG4-positive [62]; in contrast to the others, these patients were IVIG-refractory with most patients worsening after initial partial improvement and, as a group, required more extensive immunotherapy. An interesting observation in this NF155-IgG4-positive group was that 55% of them had dysphagia, dysarthria, diplopia, and ptosis while 45% had neuropathic pain due to small fiber sensory neuropathic involvement and autonomic symptoms with orthostatic intolerance, urinary incontinence, or gastrointestinal dysmobility [62].
- *LGI1/CASPR2*- and *DPPX*-*associated autoimmunities*. In several retrospective but small series, the first-line immunotherapy for those patient subsets presented with encephalitis and epilepsy has been steroids and IVIG with either encouraging or inconsistent responses [63, 64]. Although some LGI1 antibody–positive patients with epilepsy and especially pain respond to immunotherapies [25, 64], greater residual patient-rated impairment was more prominent in CASPR2 antibody patients [63]. In one retrospective series, rituximab was used in some patients with worse outcome and, although used only as second-line therapy, 3 of 4 patients reported improvement [65]. However, the noted increase of LGI1-specific plasmablasts/plasma cells in the CSF of these patients [63] justifies anti-CD19/CD20-specific immunotherapies. These retrospective data from small series in an arguably uncommon disease highlight that, as with all IgG4 antibody–mediated diseases, the response to conventional immunotherapies can be inconsistent or suboptimal necessitating increased awareness to consider rituximab early in the disease course. Importantly, need to also stress that the *LGI1/CASPR2*-associated spectrum of CNS and PNS autoimmunities is not only clinically heterogeneous but the antibodies in some of these patients may be of IgG1-3 subclass and a partial or even a very good response to IVIg or steroids may not be surprising, as observed in a very well done controlled study [64]. In DPPX-autoimmunity where the antibodies are either of IgG1 or IgG4 subclass [29], patients respond to second-line immunotherapy, most often with rituximab, exhibiting more relapses when this therapy is discontinued [29]. Case reports confirm a response to rituximab and poor benefit from other agents [66]. Nevertheless, as stated earlier, increased awareness is needed for an insidious subclass switch which may be more likely to occur in this group of patients who may either have IgG1-3 or IgG4 subclass of antibodies, exhibiting a corresponding clinical remission or worsening according to the dominant IgG subclass or a switch to the other.

### Rituximab Maintenance and Potential Value of IgG-4 Antibodies as Biomarkers: Experience from IgG4-RD Going to IgG4-ND

In several series of IgG4-RD, the risk of relapse has been lower while on rituximab maintenance, compared to only one rituximab induction therapy [2, 4, 67]; furthermore, rising serum IgG4 levels have been considered a risk factor for relapse, even though the overall value of IgG4 level alone may not always be a reliable biomarker because IgG4 titers can non-specifically increase during immune activation periods [3]. In most patients, remission seems to persist for at least 6 months and often beyond 18 months. In IgG4-RD, rituximab also shows outstanding efficacy in maintaining remission with not only associated reduction of plasmablasts and CD4 + cytotoxic T cells (CTLs) in blood and tissue, but also reduced serum markers of fibrosis and tissue myofibroblast activation [68]. The effect on fibrosis is of special interest for fibrosing diseases; it was postulated to be related to depletion of B cells that express PDGF and LOXL2 and reduced secretion of CCL4 or CCL5 which originate from B cells and limit the recruitment of monocytes and CD4 + CTLs to inflamed tissues [68]. Collectively, depletion of B cells which are also a major source of antigen presentation to CD4 + CTLs confirms the role of B cells in the pathogenesis of IgG4-RD [68].

In IgG4-ND, the IgG4 levels are irrelevant because, based on a series of CIDP patents with IgG4-nodal antibodies that we have examined, the IgG4 concentration was normal (< 1350 mg/L, the limit used for active IgG4-RD). In contrast, the IgG4-specific antibody titers can serve as key biomarkers because they correlate with disease activity, as shown for MuSK-MG [60], CIDP-associated nodopathies [20, 62], and LGI1 encephalitis where high CSF IgG4 titers strongly correlate with worse outcome [1, 63].

The role of follow-up rituximab infusions to achieve long-term remissions in IgG4-ND, although still empirical, is very reassuring. In contrast to IgG1-3-ND where patients have more labile disease and may require 2 g every 6 months or 1 g every 3 months to ensure stability [55, 56, 69], some IgG4-ND patients, at least based on Musk-MG experience, may remain free of disease for longer periods. Until controlled studies are performed and biomarkers evaluated, the main factors guiding future infusions in IgG4-ND should be the clinical status or imminent signs of early relapse. Because reduction of IgG4-MuSK antibodies coincides with clinical remission [60] and anti-nodal antibodies seem to remain low in remissions [20], the predictive value of antibody-specific titers as disease biomarkers needs to be assessed in all IgG-4-ND, as recently stressed [1]. At this point, a reliable overall biomarker still remains the reemergence of CD27 + memory B cells [55, 56, 69–71]; in one study, no MG relapses occurred when CD27 + memory B cells were below the therapeutic target, while their resurgence was associated with clinical relapses [70].

### Promising Future Therapies for IgG4-ND

The extensive somatic hypermutation shown by immunoglobulin sequencing of expanded plasmablast clones in IgG4-RD, in conjunction with the effect of rituximab in B cell depletion, provides by analogy a strong rationale for more promising effective therapies not only in IgG4-RD but also in IgG4-ND. This is especially relevant to neurology where several anti-B cell monoclonals have already been approved in autoimmune neurological diseases. Collectively, future agents, ready to explore in all aforementioned IgG4-ND, include:

1. *Anti-B cell agents*. They target CD20 by antibody-dependent cell cytotoxicity (ADCC) involving the Fcγ receptors (FcγRs), by complement-dependent cytotoxicity (CDC) or by directly inducing cellular apoptosis [72]. They are categorized as type I (rituximab, ofatumumab, and ocrelizumab) or type II (tositumomab and obinutuzumab) based on their binding properties and mechanism of action. The type I permits CD20 accumulation in lipid rafts binding fully to CD20 with high potential to induce CDC. In contrast, the type II has a half maximal CD20 binding capacity with low potential for CDC; they do however internalize less rapidly into the targeted B cells exerting a prolonged action via the FcγRIIb and induce a much higher ADCC by recruiting monocytes, neutrophils, and dendritic cells [72].
  - *Inebilizumab*, a monoclonal anti-CD19 antibody approved in neuromyelitis optica [73, 74]. Of interest, an international, multi-center trial has already begun in 160 patients with IgG4-RD [2].
  - *Ocrelizumab*, a recombinant anti-CD 20 monoclonal antibody that represents the humanized version of rituximab, approved for relapsing and progressive multiple sclerosis (MS).
  - *Ofatumumab*, a fully humanized anti-CD-20 monoclonal already, approved for relapsing–remitting MS, given monthly subcutaneously. Ofatumumab, compared to rituximab, binds not only the large loop of CD20 but also the small loop closer to the B cell membrane, leading to more effective B cell lysis [72].
  - *Obexelimab*, that targets CD19/FcγRIIB. This is especially attractive in IgG4-ND because it binds simultaneously to both CD19 and Fc**γ**RIIb, promoting internalization of CD19 in the lipid rafts [3, 72]. Because obexelimab markedly enhances the inhibitory FcγRIIB and downregulates CD19, it might prove to be more tailored to IgG-4-ND where the IgG4 have low affinity binding to FcγRIIb, exerting a more prolonged anti-B cell action affecting also antigen presentation and cytotoxic T cells.
  - *Obinutuzumab*, approved for chronic lymphocytic leukemia. Being type II anti-CD20 has increased efficiency in B cell depletion compared to type I. Because it exerts a prolonged action via the FcγRIIb, it may be attractive for the IgG-4-ND, by affecting also antigen presentation. In two rituximab-resistant patients with anti-MAG IgM antibodies and demyelinating neuropathy we have treated, no beneficial effect was however noted [75].
2. *Bruton’s tyrosine kinase inhibitors (tolebrutinib, zanubrutinib, and rilzabrutinib)*. These oral agents approved for CLL are now in phase II trials in pemphigus vulgaris [3]. They are of interest to IgG4-ND because BTK is expressed on B cells and macrophages driving inflammation and their irreversible inhibition substantially suppresses B cell activation, both in the periphery and the CNS where they easily enter. *Tolebrutinib* is now showing very promising results in a phase II trial multiple sclerosis [76].
3. *Elotuzumab*. This is a monoclonal anti-SLAMF7 antibody ready to be tested in IGg4-RD [3]. Elotuzumab that has been granted breakthrough designation by the FDA for the treatment of refractory multiple myeloma is a rational therapy for IgG-4 diseases because it targets the relevant cellular interactions between activated B cell subsets, plasmablasts, and CD4 + CTLs that all express SLAMF7 on their surfaces [3].
4. *Anti-FcRn*. The FcRn inhibitors bind to FcRn with high affinity leading to catabolism of all IgG subclasses and selective reduction of pathogenic and nonpathogenic serum IgG [52]. They can be promising agents in IgG-4-ND because they are effectively reducing pathogenic antibodies, like AChR in myasthenia gravis, greatly improving exacerbations or inducing disease stability [77, 78]. Although the FcRn inhibitors lower all IgG subclasses, the IgG3 seems more affected compared to the other three [52, 61]; in treating IgG4-ND, therefore, such a differential effect on degrading the IgG4 subclass should be of relevance in choosing the most appropriate anti-FcRn antibody. Because they do not also affect the function of memory B cells and plasma cells, their potential long-term efficacy remains uncertain. The most common agents to consider based on the ongoing trials in other antibody-mediated autoimmune neurological diseases, like myasthenia gravis, CIDP, myositis, and neuromyelitis, include:
  - *Efgartigimod*, a humanized IgG1 Fc fragment with increased binding to FcRn at neutral and acidic pH, now approved in myasthenia gravis [77]. It was noted that the IgG1-3 subclasses were equally reduced but there was a slightly smaller reduction for the IgG4 [52, 61],
  - *Rozanolixizumab*, a high affinity humanized anti-FcRn IgG4 monoclonal antibody, and
  - *Nipocalimab*, a humanized deglycosylated IgG1 monoclonal antibody that binds with high affinity to FcRn throughout the recycling pathway.

## Conclusion

The IgG4, due to its unique structural features in the hinge region, has non-inflammatory properties being functionally monovalent, unable to engage in cross-linking and internalization of targeted antigen exerting pathogenicity by blocking protein–protein interactions and signal transduction. Because IgG4 does not activate complement and cannot bind to inhibitory FcγRIIb receptor to activate cellular and complement-mediated immune responses, all the key targets inhibited by IVIg and conventional immunotherapies, IgG4-ND do not respond to IVIg like their IgG1 counterparts, but robustly respond to anti-B cell therapies. Because rituximab effectively targets the production of pathogenic IgG-4 antibodies, it should be the preferred treatment in IgG4-ND. Controlled studies with other anti-CD19/20 monoclonals, including those that also activate FcγRIIb, may be even more promising in treating IgG4-ND. Other anti-B cell agents, including Bruton’s tyrosine kinase inhibitors, and agents targeting FcRn that increase the catabolism of pathogenic antibodies are also of interest. At the molecular level, examining somatic hypermutation by immunoglobulin sequencing of expanded plasmablast clones after anti-B cell treatments my shed light in understanding IgG4-secretring B cell clones. At the clinical level, IgG4 antibody titers may become a monitoring tool in assessing disease activity and treatment response because they are reduced in remissions, increased in exacerbations, and seem to correlate with the patients’ clinical status.

## Supplementary Information

Below is the link to the electronic supplementary material.

Supplementary file1 (PDF 584 KB)
